# Phenology dictates the impact of climate change on geographic distributions of six co‐occurring North American grasshoppers

**DOI:** 10.1002/ece3.8463

**Published:** 2021-12-15

**Authors:** Nathan P. Lemoine

**Affiliations:** ^1^ Department of Biological Sciences Marquette University Milwaukee Wisconsin USA; ^2^ Department of Zoology Milwaukee Public Museum Milwaukee Wisconsin USA

**Keywords:** climate change, ecological niche model, extrapolation, grasshoppers, machine learning, orthoptera, phenology, species distribution model, spring temperature, warming

## Abstract

Throughout the last century, climate change has altered the geographic distributions of many species. Insects, in particular, vary in their ability to track changing climates, and it is likely that phenology is an important determinant of how well insects can either expand or shift their geographic distributions in response to climate change. Grasshoppers are an ideal group to test the hypothesis that phenology correlates with range expansion, given that co‐occurring confamilial, and even congeneric, species can differ in phenology. Here, I tested the hypothesis that early‐ and late‐season species should possess different range expansion potentials, as estimated by habitat suitability from ecological niche models. I used nine different modeling techniques to estimate habitat suitability of six grasshopper species of varying phenology under two climate scenarios for the year 2050. My results suggest that, of the six species examined here, early‐season species were more sensitive to climate change than late‐season species. The three early‐season species examined here might shift northward during the spring, while the modeled geographic distributions of the three late‐season species were generally constant under climate change, likely because they were pre‐adapted to hot and dry conditions. Phenology might therefore be a good predictor of how insect distributions might change in the future, but this hypothesis remains to be tested at a broader scale.

## Graphical abstract

Life history strategy might be an important determinant of how species respond to climate change. In this modeling exercise, I show that early‐season grasshoppers are more likely to expand their ranges in the future than late‐season grasshoppers.

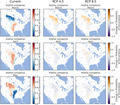

## 1 INTRODUCTION

Throughout the last century, climate change has altered the geographic distributions of many species. Insects, in particular, are rapidly expanding poleward as warming enables them to colonize previously inhospitable areas (Hickling et al., 2006). Such range shifts are best documented in lepidopterans, having been recorded in Europe (Parmesan et al., 1999), Korea (Adhikari et al., 2020), southeast Asia (Au & Bonebrake, 2019), and North America (Wilson et al., 2021), making butterflies and moths the characteristic example of poleward mobility. However, evidence of poleward shifts of other insect species is relatively sparse, documented for a handful of dragonflies, lacewings, spiders, and grasshoppers (Hickling et al., 2006), and for a few economically important agricultural pests, such as the Colorado potato beetle (Wang et al., 2017) or mountain pine beetle (de la Giroday et al., 2012). The data that do exist suggest that latitudinal shifts are quite variable among species (Beckmann et al., 2015; Chen et al., 2011). There is, as yet, no consistent pattern that explains which insect species exhibit range shifts and which do not.

Life history strategy is often invoked as a determinant of potential for range shifts (Estrada et al., 2015), but there are few phylogenetically controlled studies that contrast different life history strategies within a single clade. Orthopterans (grasshoppers, crickets, and katydids) provide an opportunity to compare sensitivity to climate change among life history strategies, given that co‐occurring grasshopper species possess a remarkable functional diversity (Deraison et al., 2015; Deraison et al., 2015; McClenaghan et al., 2015). In the United Kingdom, for example, warm‐adapted, generalist grasshoppers with high dispersal ability are the only species to have undergone range expansion (Beckmann et al., 2015). In the Great Plains of North America, grasshoppers can be broadly partitioned into two life history groups. Early‐emerging species, such as *Arphia conspersa*, *Eritettix simplex*, and *Xanthippus corallipes*, overwinter as nymphs and emerge as adults in the spring (Capinera & Sechrist, 1982). These three species reach peak abundance in April or May, several months before most other grasshopper species (Buckley et al., 2021). Late‐emerging species, such as *Arphia pseudonietana*, *Opeia obscura*, and *Phoetaliotes nebrascensis*, overwinter as eggs, hatch in early summer, and reach the adult stage by mid‐to‐late summer in July or August (Branson, 2016; Capinera & Sechrist, 1982). Given their different climatological niches, that is, cold wet spring vs. dry hot summer, and the fact that two of these species are congeners, these six species provide a phylogenetically controlled experiment for how life history might impact how species respond to climate change.

It is possible to compare how species of different life history strategies might respond to climate change using ecological niche models (ENMs). ENMs correlate occurrence records with climate and are often used to predict range expansions. For example, ENMs can identify areas at risk of invasion under future climates (Gong et al., 2020; Kistner‐Thomas, 2019) or identify high‐priority conservation targets (Garzon et al., 2021), which is critically important as the ranges of many threatened species might collapse in the near future (Lemoine, 2015). One shortcoming is that ENMs rarely account for phenology (Ingenloff & Peterson, 2021); many simply use mean annual temperature or precipitation (Booth et al., 2014; Title & Bemmels, 2018). The use of models that incorporate annual trends might miss spatiotemporal shifts in distribution because habitat suitability can change within a given year (Martinez‐Meyer et al., 2004). For example, an annual model predicted that suitable habitat for the mosquito *Aedes aegypti* should cover most of Mexico, while a temporally explicit model revealed distinct seasonal shifts in habitat suitability of *A*. *aegypti* (Peterson et al., 2005). This is also true for insects in seasonal temperate environments. The shortgrass steppe of Colorado, on average, is 8.7°C and receives 395 mm of rainfall per year. Yet early‐season grasshoppers that emerge in May experience an environment that is 22°C and receives 61 mm of rain. Late‐season grasshoppers, in contrast, emerge into an arid environment of 29°C and 40 mm of rainfall. Thus, accurate predictions in ENMs require that climatological data match life history data as closely as possible (Ingenloff & Peterson, 2021). Using mean annual temperature or precipitation might over‐ or underestimate the sensitivity of species to climate change by mischaracterizing their environmental niches.

Here, I modeled how climate change might affect intra‐annual spatiotemporal patterns of habitat suitability for six North American grasshoppers that differ in life history strategy. Specifically, I predicted that three early‐emerging species, *A*. *conspersa*, *E. simplex*, and *X. corallippes*, would favor cool, wetter temperatures. Thus, both the southern and northern boundaries of suitable habitat conditions for these three species should move northward (i.e., total range shift) and occur earlier in the year, which would predict an advancing phenology. In contrast, *A*. *pseudonietana*, *O*. *obscura*, and *P*. *nebrascensis* all emerge as adults in July and August, and therefore should have suitable habitat expand northward while maintaining the current southern boundary (i.e., range expansion), and suitable habitat should extend later into the year. To test these hypotheses, I constructed ENMs using nine separate machine learning classification techniques and predicted suitable habitat into the future for two different climate scenarios, with four general circulation models for each climate scenario used to produce an ensemble prediction.

## 2 METHODS

### 2.1 Environmental data

To construct climatic niches for each species, I downloaded WorldClim2 climate data (Fick & Hijmans, 2017), which is an interpolated climate dataset covering the years 1970–2000. As I was specifically examining phenological patterns, I used monthly data at a 5 arc‐minute resolution. The use of monthly data restricted the environmental variables to average monthly precipitation, average monthly temperature, minimum monthly temperature, and maximum monthly temperature, as other WorldClim2 variables are either seasonal aggregates or unavailable at monthly time steps. Given the extremely high correlation among temperature variables (*r* > .90 for all temperature combinations, Table S1), I used only mean monthly precipitation (mPPT) and mean minimum monthly temperature (mT_min_) for all subsequent analyses.

### 2.2 Species occurrence records

I constructed ENMs for six grasshopper species: *A. conspersa*, *A. pseudonietana*, *E. simplex*, *O. obscura*, *P. nebrascensis*, and *X*. *corallipes*. These species are all common throughout North American grasslands and cluster into early (*A*. *conspersa*, *E*. *simplex*, and *X*. *corallipes*) and late (*A*. *pseudoneitana*, *O*. *obscura*, and *P*. *nebrascensis*) phenological life histories (Capinera & Sechrist, 1982). Furthermore, these six species possessed suitable numbers of occurrence records; records for most other North American grasshopper species were too limited to accurately construct ENMs. I downloaded species occurrence records from the Global Biodiversity Information Facility (GBIF) in April, 2018. In total, there were 9091 georeferenced locations [*A*. *conspersa*: 2117 (https://doi.org/10.15468/dl.yiryb8); *A*. *pseudonietana*: 788 (https://doi.org/10.15468/dl.gekxzl); *E*. *simplex*: 2441 (https://doi.org/10.15468/dl.clvt05); *O*. *obscura*: 659 (https://doi.org/10.15468/dl.46qhao); *P*. *nebrascensis*: 634 (https://doi.org/10.15468/dl.tazqk5); and *X*. *corallipes*: 2452 (https://doi.org/10.15468/dl.woyqdj)]. Date ranges for the six species are as follows: *A*. *conspersa*: 1899–2013; *A*. *pseudonietana*: 1885–2013; *E*. *simplex*: 1912–2017; *O*. *obscura*: 1905–2012; *P*. *nebrascensis*: 1889–2013; and *X*. *corallipes*: 1903–2017. Accession data are available on figshare (10.6084/m9.figshare.14411048), and distribution maps of the raw data are available in Figure S1.

### 2.3 Data cleaning, filtering, and pseudoabsences

I cleaned GBIF records following a standard pipeline (Feng et al., 2019; Zurell et al., 2020). First, I dropped any records with null values for latitude, longitude, month, or year. Next, I removed records with a “0” for latitude or longitude. I then dropped any observations that had coordinates identical to those of a US state capital city to within 0.01 decimal degrees, and also dropped any duplicate geographic coordinates except for those observations in different months and years. Once this pre‐screening was complete, I visually checked distribution maps and removed any erroneous observations. During visual checks, I removed two observations of *E*. *simplex* in the southeastern United States, as well as any observations falling below 20°N, which were outside the range of environmental layers. These data cleaning steps reduced the number of records to *A*. *conspersa*: 1255, *A. pseudonietana*: 309, *E*. *simplex*: 1830, *O*. *obscura*: 315, *P*. *nebrascensis*: 372, and *X*. *corallipes*: 1746.

I then filtered data to remove pseudoreplicates in environmental space. Although many studies advocate spatial filtering, I instead filtered observations on the basis of environmental similarity. Such environmental filtering has shown to be more robust, less biased, and more accurate than spatial filtering (Varela et al., 2014). For the environmental filter, I created 50 evenly spaced bins along both mPPT and mT_min_, and dropped any duplicate observations within a grid cell (Figure S2). By removing environmental pseudoreplicates, filtering further reduced the number of observations to *A*. *conspersa*: 124, *A*. *pseudonietana*: 66, *E*. *simplex*: 85, *O*. *obscura*: 36, *P*. *nebrascensis*: 41, and *X*. *corallipes*: 75. The geographic distributions of these samples are identical to the raw data, albeit with no duplicates within a given set of coordinates (Figure S3). The final dataset included observations from year 1885 to 2017; however, >50% of the observations were more recent than 1970 and over a third of the observations were from the year 2000 or later.

Due to the temporal aspect of the hypotheses tested here, I used a phenological approach to generating pseudoabsences (Ingenloff & Peterson, 2021). Briefly, for each species, I calculated the number of observations falling within each month. I then generated the same number of pseudoabsences from the mPPT and mT_min_ for that month. The end product was the same number of observations and pseudoabsences for each species within each month. I chose to use equal numbers of pseudoabsences because a 1:1 ratio of observations:pseudoabsences performs the best for many classification models (Barbet‐Massin et al., 2012). I used a simple random pattern, rather than a gridded or weighted approach, because multiple studies demonstrated that simple random pseudoabsences perform at least as well as weighted or stratified pseudoabsences, especially for some of the classification methods used here (Barbet‐Massin et al., 2012; Hanberry et al., 2012).

### 2.4 Ecological niche models

ENMs use correlative approaches to summarize the climatic niche of a species. There is a large degree of uncertainty in ENMs, including uncertainty due to presence‐only sampling, spatial biases, and in climate models. Perhaps the largest source of uncertainty is among modeling techniques (Araújo et al., 2005). Different methods make different assumptions, and these assumptions often result in variable ENM projections (Aguirre‐Gutiérrez et al., 2013). Here, I account for methodological uncertainty by using nine different machine learning approaches to construct ENMs:

1. *Logistic regression (GLM)*: Logistic regression is a standard technique in many ENM studies. GLM proceeds by regressing the binary response variable (presence/pseudoabsence) against the environmental predictions mPPT and mT_min_. Here, I used an additive model structure:

$$y∼{\text{logit}}^{‐1}\left(z\right)$$

$$z=\beta_{0}+\beta_{1}\text{mPPT}+\beta_{2}{\text{mT}}_{\min}$$

which did not include an interaction between mPPT and mT_min_. In this model, *y* is presence/pseudoabsence (1/0) and *z* is the log odds of occurrence (i.e., logit transformation).

2. *K*‐*Neighbors Classifier (KNC)*: A KNC uses a simple “vote‐counting” method to assign a point to a class. Essentially, an unknown point (test data) is mapped into environmental space with training data. The algorithm counts the *n* nearest neighbors and assigns the test point to the class with the majority or plurality of neighbors. The output can be converted into a probability by counting the fraction of *n* points belonging to a given class. For the model here, I used *n *= 5 equally weighted neighboring points, and the distances between training points and the test points in environmental space were determined via Euclidean distance.
3. *Gaussian Process Classifier (GPC)*: Gaussian process models treat data as arriving from a multivariate distribution, generated by an unknown function:

$$f\left(x\right)∼\text{GP}\left(m\left(x\right),K\left(x,x^{\prime }\right)\right)$$

where *f*(*x*) is the function describing the variability of *x* in space, *m*(*x*) is the mean function, and *K*(*x*, *x*′) is the kernel/covariance function. Because the kernels allow for covariance among observations that varies with the distance of observations, continuous Gaussian process models are popular for time series and spatial modeling, where they are known as “kriging” (Brahim‐Belhouari & Bermak, 2004; Roberts et al., 2013). GPCs extend Gaussian process models to a binomial response using latent variables, much like logistic regression:

$$f\left(x\right)={\text{logit}}^{‐1}\left(z\left(x\right)\right)$$

$$z\left(x\right)∼\text{GP}\left(m\left(x\right),K\left(x,x^{\prime }\right)\right)$$

where *z*(*x*) is a latent variable achieved by the logistic transformation of pseudoabsence (0) and presence (1) data. In practice, we often assume a constant mean:

$$z\left(x\right)∼\text{GP}\left(0,K\left(x,x^{\prime }\right)\right)$$

such that the kernel choice dictates the shape of the function. Researchers have advocated GPCs for ENMs because they are often more accurate than other classification methods, such as boosted regression trees, generalized additive models, and generalized linear models (Golding & Purse, 2016). Here, I constructed ENMs from GPCs using the radial basis function:

$$K\left(x,x^{\prime }\right)=\alpha^{2}\exp\left(‐0.5l^{‐2}{\left(x‐x^{\prime }\right)}^{2}\right)$$

where *α* is a scaling parameter determining the magnitude of process noise and *l* is a length parameter that determines the smoothness of the function.

4. *Decision Tree Classifier (DTC)*: DTCs are nonparametric, supervised machine learning techniques that construct decision trees using if/then rules from training data in order to infer the class of the test points. Essentially, decision trees split the data into groups then conduct logistic regressions to classify the training data. The split with the highest predictive ability is taken as the first decision criteria to generate two new groupings within the next level of the tree. The procedure proceeds iteratively within each grouping until a maximum tree depth is achieved. These models are simple, fast, and nonlinear, but can be prone to overfitting, particularly if a tree is too deep. For the model here, I used the Gini criteria to evaluate the quality of a given split, with a maximum tree depth of five levels. I required each group to have a minimum of two samples.
5. *Random Forest Classifier (RFC)*: An RFC is a “meta”‐classifier that constructs a number of DTCs from random subsamples of the training data and averages the outputs. For the RFC here, I generated 100 random DTCs using the same Gini criteria.
6. *Artificial Neural Network (ANN)*: ANNs with multilevel perceptrons approximate the way human brains process information by allowing computing nodes, called neurons, to process and share information to inform an output. ANNs consist of three layers of nodes: input nodes, hidden process nodes, and output nodes. The input layer contains nodes for each feature (i.e., explanatory variable), hidden process nodes combine features with a weighted linear function, and an output function uses a nonlinear function to transform the hidden process nodes into a binary or continuous response. Several authors have advocated using ANNs for ENMs (Maravelias et al., 2003), in particular because they outperform many other methods for constructing ENMs, such as classification trees, generalized linear models, generalized additive models, and spatial interpolators (Segurado & Araújo, 2004). I trained the linear weights using a stochastic gradient optimizer, and the nodes were translated into a real output using the rectified linear unit function max(0, *x*). The ANN here had one hidden layer with 100 nodes, and a regularization parameter *α* = 1.
7. *Ada Boost Classifier (ABC)*: The ABC is similar to RFC, in that it relies on multiple DTCs. However, whereas RFCs generate 100 random DTCs and then average the outputs, ABCs proceed iteratively, repeatedly fitting the same DTC on the training data but with the weights of incorrect cases adjusted so the classifier focuses on more difficult cases. I used the SAMME.R algorithm, stopping at a maximum of 50 iterations. The SAMME.R algorithm is an updating algorithm that uses the probabilities of belonging to each class for each point as weights in an exponential loss function used to assess model fit (Pedregosa et al., 2011).
8. *Naïve Bayesian Classifier (NBC)*: NBCs are simple classifiers based on Bayes' rule. Bayes' rule can calculate the probability that a given map pixel should belong to a class *k* (i.e., present/absent) as:

$$p\left(k|x\right)=p\left(x|k\right)p\left(k\right)/p\left(x\right)$$

where *x* is the environmental variable, *p*(*k*|*x*) is the probability that a pixel of a given environment *x* belongs to class *k*, *p*(*k*) is the prior probability of belonging to class *k*, and *p*(*x*) is the probability of the environmental variable occurring in the model. For example, imagine classifying whether a pixel should be suitable habitat for a bird (*k* = present), depending on whether it is forested or not. In this case, *p*(*x*|*k*) is the probability that a pixel is forest given that a bird is present, or the proportion of times a bird was observed in forests, *p*(*k*) is the proportion of sightings of the bird throughout the entire dataset, and *p*(*x*) is the proportion of pixels that are forested. This example has a discrete predictor, but Gaussian NBCs extend classification to continuous predictors, such as temperature, by using the Gaussian density distribution to calculate the likelihood of a given temperature given an observation of present or absent:

$$p\left(x|k\right)=\left(1/\text{sqrt}\left(2\text{pi}\sigma_{k}^{2}\right)\right)e\left[‐0.5{\left(x‐\mu_{k}\right)}^{2}/\sigma_{k}^{2}\right]$$

In this case, the probability of a bird being present at a given temperature is

$$p\left(k|x\right)\propto p\left(x|k\right)p\left(k\right)$$

This method can be extended to multiple predictors by:

$$p\left(k|x_{1},x_{2},\ldots ,x_{n}\right)\propto p\left(x_{1}|k\right)p\left(x_{2}|k\right)\ldots p\left(x_{n}|k\right)p\left(k\right)$$

Gaussian NBCs, along with the other methods here, can be used as a classification algorithm to model species niches (Guo & Liu, 2010). A drawback of this method is that it assumes independence of the features, but it has been shown to be an accurate method for constructing ENMs (Guo & Liu, 2010).

9. *Quadratic Discriminant Analysis (QDA)*: QDA is a generalization of linear discriminant analysis, and also of NBCs. As with NBCs, QDA uses Bayes' rule to maximize the posterior probability *p*(*k*|*x*). There are two big differences between NBCs and QDAs. The first difference is that NBCs assume the predictors are conditionally independent, while QDA allows for the predictors to be correlated:

$$p\left(x|k\right)=\left\{1/\left[{\left(2\text{pi}\right)}^{d/2}\Sigma^{0.5}\right]\;\right\}e\left[‐0.5{\left(x‐\mu \right)}^{\prime }\Sigma^{‐1}\left(x‐\mu \right)\right]$$

where *d* is the number of features and Σ is the covariance matrix of the features. When the classes are assumed to have the same Σ, and also that Σ is diagonal (i.e., features are independent), this formula reduces to an NBC. If the *k* classes have the same Σ, but Σ is not diagonal, this formula reduces to linear discriminant analysis, as in the previous equation. If the classes are allowed to have separate covariances Σ*_k_*:

$$p\left(x|k\right)=\left\{1/\left[{\left(2\text{pi}\right)}^{d/2}\Sigma_{k}^{0.5}\right]\;\right\}e\left[‐0.5{\left(x‐\mu \right)}^{\prime }\Sigma^{‐1}\left(x‐\mu \right)\right]$$

then the formula is QDA. QDA is attractive because discriminant analyses typically perform well and require no hyperparameters to tune. Parameters are fit to training data, and then the resulting model is used to estimate the test data.

Prior to analyses, both mPPT and mT_min_ were standardized to *N*(0, 1) distributions to improve model fitting. Data were then split into training and test groups containing 66% and 33% of the data, respectively. Data were split in a stratified manner to ensure equal proportions of presences/pseudoabsences in both the training and test data. Models were fit to the training data, and then tested for goodness of fit on the test data using the area under receiver operating characteristic curves (AUC‐ROC). AUC‐ROC scores for each of the nine models were then averaged to produce an “ensemble AUC‐ROC” (Araújo et al., 2005).

For every species, I projected the current distribution throughout every month of the year based on WorldClim2 monthly data for mPPT and mT_min_ at 5 arc‐minute resolution (Fick & Hijmans, 2017). Model outputs were clipped to North American grasslands based on the US EPA Ecoregions Level 1 (Ecoregion 9.0 – Great Plains). After clipping, predictions from each of the nine modeling techniques were averaged (unweighted) to generate a single ensemble prediction for each species/month combination (Araújo et al., 2005). To simplify visualization, I chose to display the months of March, April, and May for early‐season species, and July, August, and September for the late‐season species. Graphs for all other months are available on Figshare (see *Data Accessibility*).

### 2.5 Climate change projections

I accounted for uncertainty in climate projections in two ways. First, I projected ecological niches into 2050 for intermediate and unconstrained representative concentration pathways (RCPs). The intermediate scenario was RCP 4.5, which assumes that CO_2_ emissions peak in 2040 and then decline, CH_4_ emissions stop increasing by 2050, and SO_2_ concentrations steadily decline from the present day (IPCC, 2014). As a result, average global temperatures increase by 2.5°C by 2100. The severe pathway was RCP 8.5, which assumes continuous increases in emissions throughout the 21st century, resulting in a 5°C increase in global average temperatures by 2100 (IPCC, 2014). The RCP 8.5 scenario is generally considered unrealistic, as it does not account for either biological or political feedbacks to mitigate emissions (Peters & Hausfather, 2020). However, the RCP 8.5 scenario is still useful as a “worst‐case” baseline.

The final source of uncertainty is in general circulation model (GCM) projections themselves; each GCM uses different forcings and parameters, leading to considerable variability among model outputs. To account for model uncertainty, I projected ENMs into future climates using four different GCMs: BCC‐CCSM‐1‐1 (Wu et al., 2014), CCSM4 (Meehl et al., 2012), IPSL‐CM5A‐LR (Dufresne et al., 2013), and MIROC5 (Watanabe et al., 2010). For each GCM, I estimated habitat suitability of each species, in every month, for each of the nine modeling techniques. I averaged the outputs from each of the nine modeling techniques to produce a single, ensemble estimate for each species/month/GCM combination. I then averaged the four GCM ensemble projections (i.e., four stacked models) into a single ensemble prediction of future habitat suitability in each month for each species. As above, RCP projections were trimmed to North American Grasslands using EPA EcoRegions Level 1 – 9.0 – Great Plains. GCMs are available for download from the Livermore National Lab.

## 3 RESULTS

**FIGURE 1:**
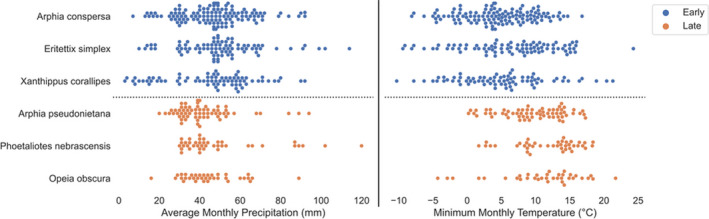
Early‐season grasshopper species were characterized by wetter, cooler conditions than late‐season species. This graph shows mPPT and mT_min_ for each observation of the cleaned, environmentally filtered data. Each point is a unique observation

Co‐occurring grasshopper species possessed different climatological niches, depending on phenology. Early‐season species (*A*. *conspersa*, *E*. *simplex*, and *X*. *corallipes*) occurred in wetter, cooler conditions common in March through May, while late‐season species (*A*. *pseudonietana*, *O*. *obscura*, and *P*. *nebrascensis*) occupied warmer, drier climate niches prevalent in July, August, and September (Figure 1). When reconstructing these climate niches, modeling algorithms varied in their performance, although models performed similarly within a species (Table 1). That is, models within a species produced similar AUC‐ROC scores (*SD* < 0.05), with the exception of *O*. *obscura*, where GPCs, NBCs, and QDAs performed exceptionally well (Table 1). These models only performed well for *O*. *obscura*, however, and no modeling technique consistently outperformed or underperformed all others across every species. For example, despite the excellent fit of NBCs and QDAs for *O*. *obscura*, these two methods provided among the poorest fits for *P*. *nebrascensis* (Table 1). Unweighted model averaging eliminated much of this variability and resulted in ensemble model fits that were consistent (AUC‐ROC scores between 0.7 and 0.8) across all species, thereby eliminating the vagaries of any single classifier.

**TABLE 1:** AUC‐ROC estimates for each model type for each of the six species

	Early species			Late species		
	*Arphia conspersa*	*Eritettix simplex*	*Xanthippus corallipes*	*Arphia pseudonietana*	*Opeia obscura*	*Phoetaliotes nebrascensis*
GLM	0.76	0.68	0.62	0.78	0.79	0.74
KNC	0.80	0.81	0.75	0.66	0.86	0.74
GPC	0.83	0.71	0.68	0.81	0.92	0.60
DTC	0.77	0.70	0.62	0.77	0.64	0.74
RFC	0.83	0.77	0.71	0.79	0.81	0.78
ANN	0.85	0.74	0.68	0.84	0.82	0.65
ABC	0.76	0.72	0.66	0.71	0.72	0.65
NBC	0.82	0.71	0.69	0.85	0.97	0.64
QDA	0.82	0.75	0.68	0.85	0.99	0.66
Ensemble	0.81 ± 0.03	0.73 ± 0.04	0.68 ± 0.04	0.78 ± 0.07	0.84 ± 0.11	0.69 ± 0.06

Note

Ensemble shows the average ±1 *SD* of the nine models.

Abbreviations: ABC, Ada boost classifier; ANN, Artificial neural network; DTC, Decision tree classifier; GLM, logistic regression; GPC, Gaussian process classifier; KNC, K‐nearest neighbors; NBC, Naive Bayesian classifier; QDA, Quadratic discriminant analysis; RFC, Random forest classifier.

**FIGURE 2:**
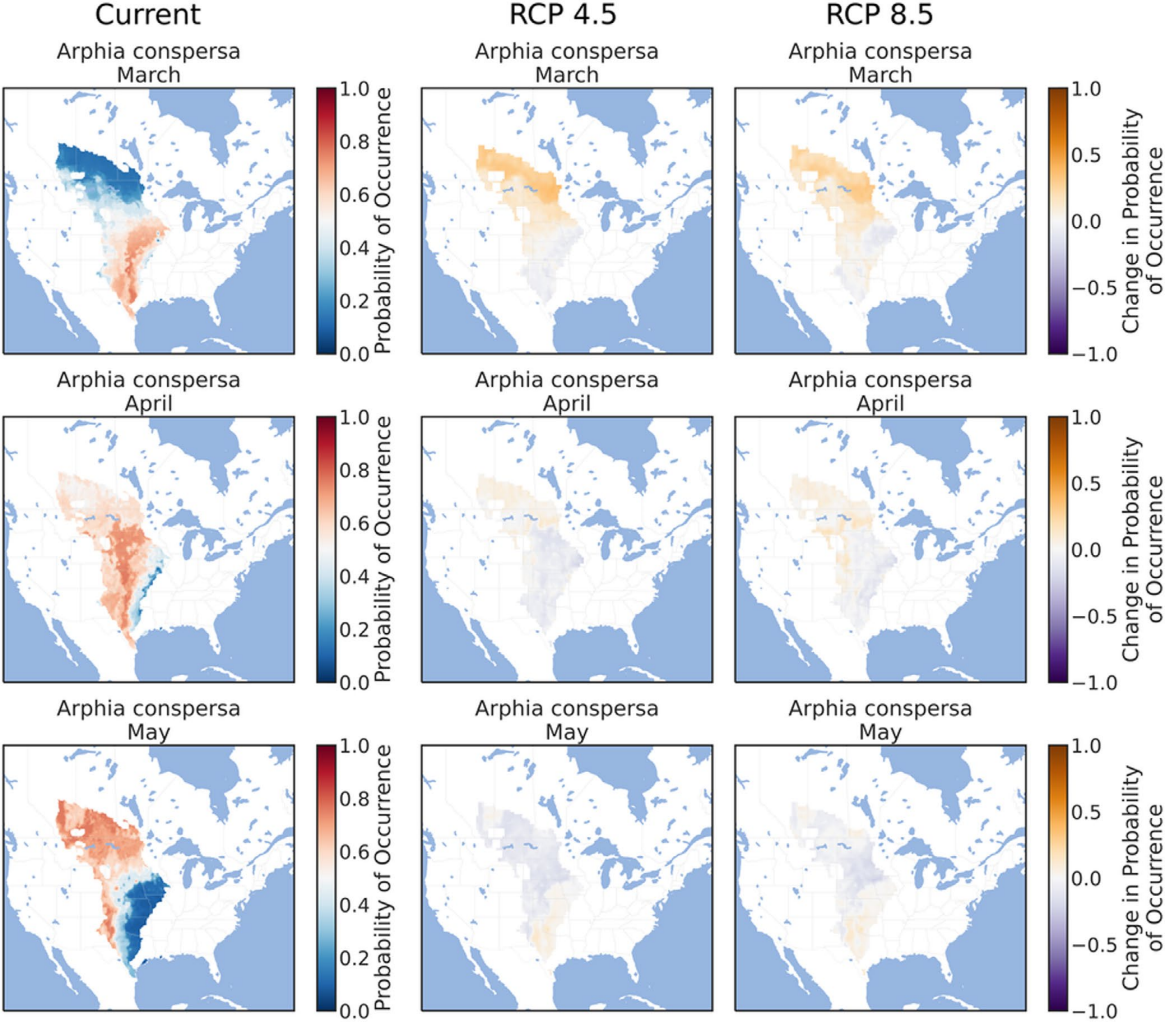
Predicted, current distribution of the early‐season species *Arphia conspersa* in March, April, and May throughout the Great Plains of North America under current conditions, RCP 4.5, and RCP 8.5. Predictions are the ensemble/stacked averages from the nine different classifiers. The color palette was chosen so that regions where absence is more likely than presence (probability of occurrence <0.5) are shaded in blue, while regions where presence is more likely than absence (probability of occurrence >0.5) are shaded in reds. Regions where presence and absence are equiprobable (probability of occurrence ~0.5) are shaded in whites/greys. Panels for RCP 4.5 and RCP 8.5 show the change in habitat suitability across each month, with orange regions denoting an increase in habitat suitability, and purple regions denoting a decrease in habitat suitability. Raw probabilities for each climate scenario are given in the supplemental figures

**FIGURE 3:**
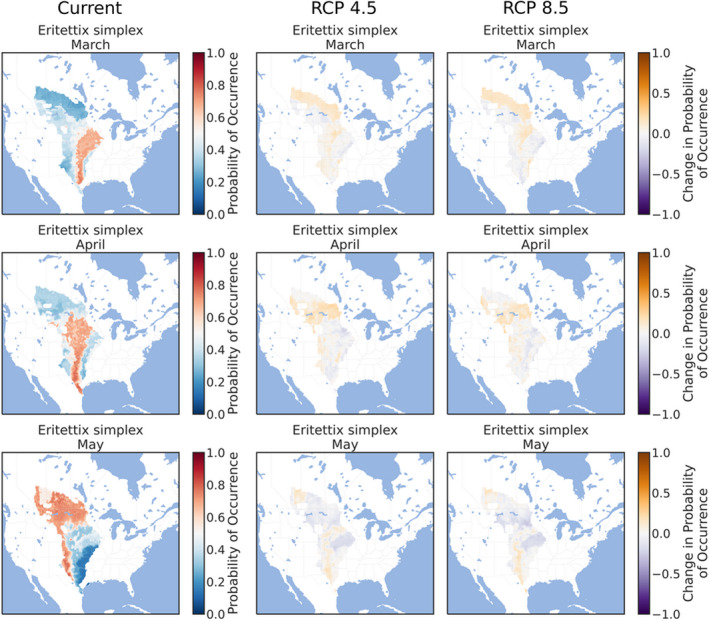
Predicted, current distribution of the early‐season species *Eritettix simplex* in March, April, and May throughout the Great Plains of North America under current conditions, RCP 4.5, and RCP 8.5. Predictions are the ensemble/stacked averages from the nine different classifiers. The color palette was chosen so that regions where absence is more likely than presence (probability of occurrence <0.5) are shaded in blue, while regions where presence is more likely than absence (probability of occurrence >0.5) are shaded in reds. Regions where presence and absence are equiprobable (probability of occurrence ~0.5) are shaded in whites/greys. Panels for RCP 4.5 and RCP 8.5 show the change in habitat suitability across each month, with orange regions denoting an increase in habitat suitability, and purple regions denoting a decrease in habitat suitability. Raw probabilities for each climate scenario are given in the supplemental figures

**FIGURE 4:**
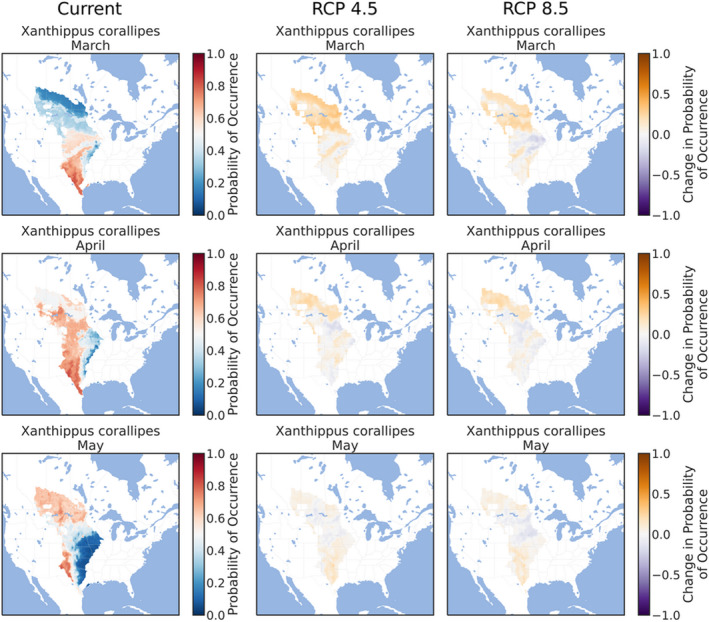
Predicted, current distribution of the early‐season species *Xanthippus corallipes* in March, April, and May throughout the Great Plains of North America under current conditions, RCP 4.5, and RCP 8.5. Predictions are the ensemble/stacked averages from the nine different classifiers. The color palette was chosen so that regions where absence is more likely than presence (probability of occurrence <0.5) are shaded in blue, while regions where presence is more likely than absence (probability of occurrence >0.5) are shaded in reds. Regions where presence and absence are equiprobable (probability of occurrence ~0.5) are shaded in whites/greys. Panels for RCP 4.5 and RCP 8.5 show the change in habitat suitability across each month, with orange regions denoting an increase in habitat suitability, and purple regions denoting a decrease in habitat suitability. Raw probabilities for each climate scenario are given in the supplemental figures

**FIGURE 5:**
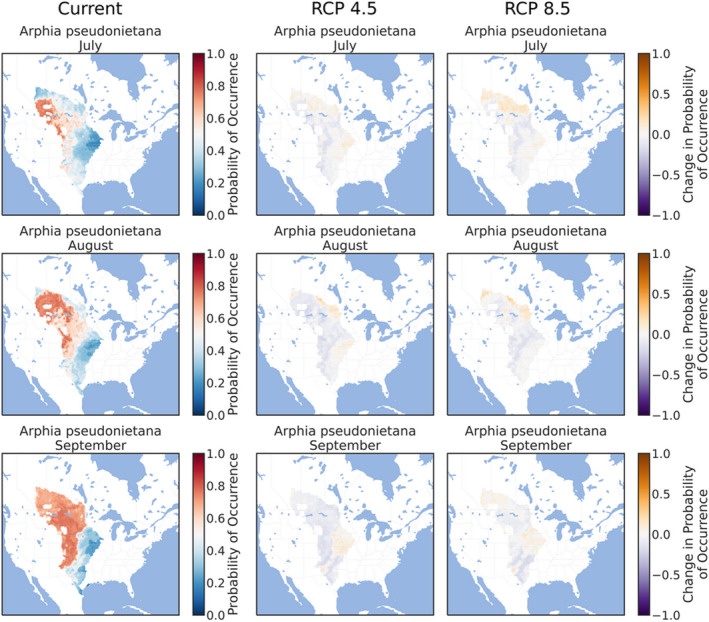
Predicted, current distribution of the early‐season species *Arphia pseudonietana* in March, April, and May throughout the Great Plains of North America under current conditions, RCP 4.5, and RCP 8.5. Predictions are the ensemble/stacked averages from the nine different classifiers. The color palette was chosen so that regions where absence is more likely than presence (probability of occurrence <0.5) are shaded in blue, while regions where presence is more likely than absence (probability of occurrence >0.5) are shaded in reds. Regions where presence and absence are equiprobable (probability of occurrence ~0.5) are shaded in whites/greys. Panels for RCP 4.5 and RCP 8.5 show the change in habitat suitability across each month, with orange regions denoting an increase in habitat suitability, and purple regions denoting a decrease in habitat suitability. Raw probabilities for each climate scenario are given in the supplemental figures

**FIGURE 6:**
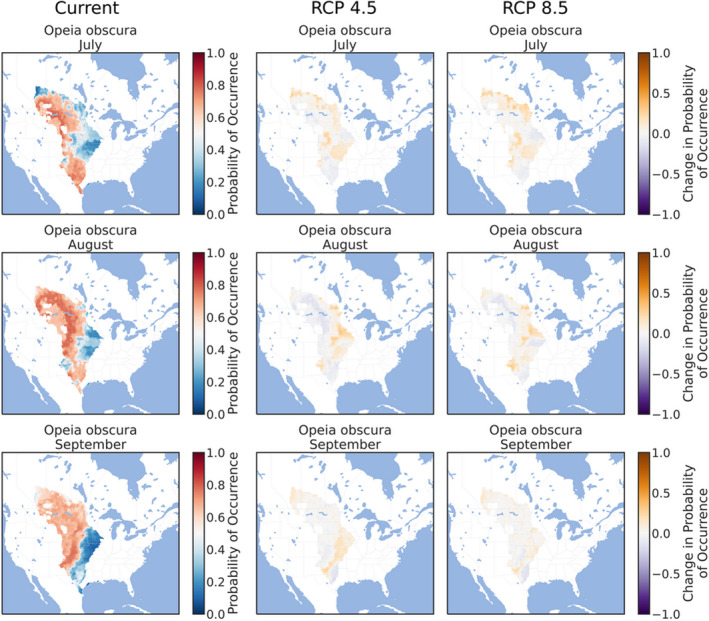
Predicted, current distribution of the early‐season species *Opeia obscura* in March, April, and May throughout the Great Plains of North America under current conditions, RCP 4.5, and RCP 8.5. Predictions are the ensemble/stacked averages from the nine different classifiers. The color palette was chosen so that regions where absence is more likely than presence (probability of occurrence <0.5) are shaded in blue, while regions where presence is more likely than absence (probability of occurrence >0.5) are shaded in reds. Regions where presence and absence are equiprobable (probability of occurrence ~0.5) are shaded in whites/greys. Panels for RCP 4.5 and RCP 8.5 show the change in habitat suitability across each month, with orange regions denoting an increase in habitat suitability, and purple regions denoting a decrease in habitat suitability. Raw probabilities for each climate scenario are given in the supplemental figures

**FIGURE 7:**
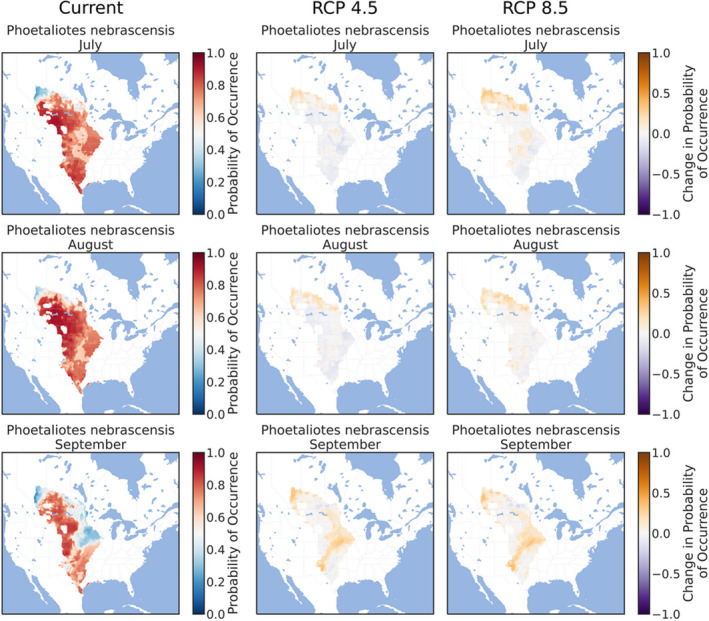
Predicted, current distribution of the early‐season species *Phoetaliotes nebrascensis* in March, April, and May throughout the Great Plains of North America under current conditions, RCP 4.5, and RCP 8.5. Predictions are the ensemble/stacked averages from the nine different classifiers. The color palette was chosen so that regions where absence is more likely than presence (probability of occurrence <0.5) are shaded in blue, while regions where presence is more likely than absence (probability of occurrence >0.5) are shaded in reds. Regions where presence and absence are equiprobable (probability of occurrence ~0.5) are shaded in whites/greys. Panels for RCP 4.5 and RCP 8.5 show the change in habitat suitability across each month, with orange regions denoting an increase in habitat suitability, and purple regions denoting a decrease in habitat suitability. Raw probabilities for each climate scenario are given in the supplemental figures

The ensemble ENMs successfully replicated the expected patterns of species' phenologies in current climate conditions. The three early‐season grasshoppers were prevalent throughout the southern and eastern Great Plains in March (Figures 2, 3, 4), although *X*. *corallipes* appeared more constrained to New Mexico, western Texas, and southeastern Colorado than either *A*. *conspersa* or *E*. *simplex* (Figure 4). By April, all three species were predicted to occur throughout the Great Plains, except for Canada and the eastern portion encompassing Iowa and eastern Kansas (Figures 2, 3, 4). By May, grasslands south of Montana and North Dakota became unsuitable, except for a north–south band along the Rocky Mountains (Figures 2, 3, 4). Likewise, phenological ENMs of the three late‐species grasshoppers examined here also generally followed my hypotheses, but with more interspecific variability than demonstrated by the three early‐season species. The red‐winged grasshopper, *A*. *pseudonietana*, was confined to Montana, Alberta, and Wyoming in July (Figure 5), whereas suitable habitat for *O*. *obscura* extended throughout the Great Plains, except for Kansas, most of Oklahoma, and Iowa (Figure 6). ENMs predicted that *P*. *nebrascensis* should be found throughout the entire Great Plains in July and August (Figure 7). By September, the southern range limit of *A*. *conspersa* had extended to New Mexico and the Texas panhandle (Figure 5). The range of *O*. *obscura* in September was generally the same as in July and August (Figure 6), and the geographic distribution of *P*. *nebrascensis* in September excluded Iowa, eastern Nebraska and South Dakota, most of North Dakota, and the northern edge of the Great Plains in Alberta (Figure 7).

I expected that climate change would cause suitable habit to expand northward in March, April, and May for the three early‐season grasshoppers. This is equivalent to both a northern range expansion but also to advanced phenology in those northern locations that become suitable earlier in the year. As predicted, all three early‐season grasshoppers generally showed northern range expansions in the summer months. For *A*. *conspersa*, the northern range limit in March moved from Iowa and Nebraska to North Dakota and Montana under both RCP 4.5 and RCP 8.5 (Figure 2). By April and May, however, the range of *A*. *conspersa* was generally unaffected by climate change, as this species already extends to the northern edge of North American grasslands (Figure 2). A similar trend was predicted for *X*. *corallipes* (Figure 4), while the range of *E*. *simplex* was unchanged for either RCP 4.5 or RCP 8.5 (Figure 3). Contrary to my predictions, ENMs did not predict a southern range contraction for any of the early‐season species, meaning that these species might see an expansion of suitable habitat area, rather than a range shift of both northern and southern boundaries, under climate change.

In contrast to the three early‐season grasshoppers, the geographic distributions of all three late‐season species were relatively stable under both RCP 4.5 and RCP 8.5 climate scenarios, refuting my hypothesis that these species should demonstrate range expansions. For example, the geographic distribution of *A*. *pseudonietana*, across all months, in both RCP 4.5 and RCP 8.5 was almost identical to the distribution of current climates (Figure 5). Similarly, climate change had little effect on the modeled distribution of *O*. *obscura*, except for a slight northward and eastward expansion of suitable habitat in July and August (Figure 6). Only *P*. *nebrascensis* conformed to my hypothesis with northward range expansions in all months under climate change (Figure 7). However, as *P*. *nebrascensis* covers most of the Great Plains under current conditions, the northward expansion was relatively minor and extended into small regions in central Alberta (Figure 7). Otherwise, suitable habitat for *P*. *nebrascensis* expanded into the eastern portions of the Great Plains (Figure 7).

**FIGURE 8:**
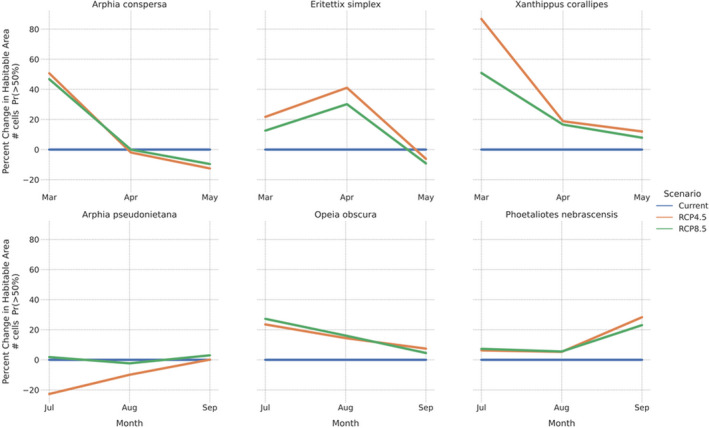
Percent change in suitable habitat area for each species under two climate scenarios. Suitable habitat area was calculated as the number of grid cells where the probability of occurrence was greater than 50%

Examining range expansions as an increase in suitable habitat area highlighted the difference between the early‐ and late‐season grasshopper species examined here. Early‐season species, *A*. *conspersa*, *E*. *simplex*, and *X*. *corallipes*, generally showed a 20–80% increase in suitable habitat area during the spring months (Figure 8), much of which was driven by northern range expansions. The three late‐season species demonstrated a lesser degree of range expansion; suitable habitat for *O*. *obscura* and *P*. *nebrascensis* increased by <20% in most months, while *A*. *pseudonietana* showed evidence for range collapse under RCP 4.5 (Figure 8). As described above, much of the increase in suitable habitat for *P*. *nebrascensis* was a longitudinal expansion, rather than a latitudinal shift (Figure 7).

## 4 DISCUSSION

As climate change alters the fundamental abiotic template of most ecosystems, many species are tracking favorable climates northward or to higher elevations. Yet, species vary in their ability to follow suitable climates (Beckmann et al., 2015; Chen et al., 2011). While life history characteristics like dispersal undoubtedly play a role in the capacity for range expansion (Beckmann et al., 2015), I hypothesized that the three early‐season species examined here would shift poleward while late‐season species would have relatively stable geographic distributions due to shifts in habitat suitability in future climates. In testing these hypotheses, I was able to partially confirm my hypotheses. The three early‐season species exhibited range expansions via a poleward shift of the northern range limit while maintaining southern range limits, while the three late‐season species appeared largely unaffected by climate change. Thus, it appears that spatially co‐occurring species might exhibit different responses to climate change based on phenology, and my work highlights the need to account for emergence phenology in species distribution modeling.

Phenological shifts are common responses to climate change for both plants and insects. In plants, warming often advances emergence and flowering dates (Price & Waser, 1998; Wolkovich et al., 2012). However, not all plant species advance their phenology with warming; the phenological response to warming appears to largely depend on plant life history. Spring species that flower early often advance their phenology, sometimes by several weeks, while species that flower in fall can delay their phenology (Sherry et al., 2007). Like plants, insects also advance their emergence dates (Ellwood et al., 2012). Yet, few studies have tested whether phenology (i.e., overwintering state) influences how insects alter geographic distributions in response to climate change (but see Poyry et al., 2009). Grasshoppers are an ideal system to test for such possibilities because co‐occurring species, indeed even co‐occurring congeners as in the case of *Arphia*, possess early and late phenologies (Capinera & Sechrist, 1982), providing the opportunity for phylogenetically controlled tests of range expansion.

My study suggests that, as with plants, insect emergence phenology might be an important predictor of how insects respond to climate change. In this study, three early‐season grasshopper species did not advance their phenology across their entire range, but only in the northernmost regions of the Great Plains. Viewed spatially, this pattern amounts to a northern range expansion in early spring, and viewed temporally, it amounts to an advanced phenology in northern areas. However, late‐season species that share the same geographic distribution as early‐season species might shift their distributions less in response to climate change. This is likely because climate change will make much of North America both warmer and drier (Greve et al., 2014; Sheffield & Wood, 2008), an environment to which late‐season grasshoppers are already adapted. Importantly, no species here showed a range collapse; all grasshopper species examined here are predicted to maintain, if not expand, their current range size. This matches predictions of many other insects (Au & Bonebrake, 2019; de la Giroday et al., 2012; Wilson et al., 2021), and suggests that climate change might not directly precipitate the decline in insect abundances.

It is also likely that grasshopper populations vary in their sensitivity to climate change depending on latitude. Latitudinal temperature variation plays a strong role in grasshopper life history, with northern populations being often being smaller, developing slower, and having lower egg viability than warmer, southern populations (Telfer & Hassall, 1999). There is little information on how grasshopper phenology varies with latitude, but numerous studies have documented delayed eclosion/emergence dates for grasshoppers in cold, high‐elevation populations (Buckley et al., 2021; Nufio & Buckley, 2019). Northern populations might therefore have delayed phenologies due to a slower accumulation of growing degree days, and these populations might be primed to advance their phenologies more strongly than southern populations where climate warming will be weaker and growing degree days already accumulate rapidly (Diffenbaugh & Giorgi, 2012). Indeed, this pattern is already predicted by the ENMs here, which showed phenological advances only in the most northerly populations.

One important caveat is that the ENMs reported here account for only climate and do not include biotic interactions. Although ENMs here predicted range expansions, grasshoppers could experience a large decline in range size with continued disappearance of grasslands, caused by either climate or land use change. A recent study from Germany found that land use change and habitat loss were major factors responsible for a decades‐long collapse of insect populations (Hallmann et al., 2017). In ENMs, habitat availability can be the strongest determinant of insect distributions in both current and future climates (Lemoine, 2015). Thus, although the ENMs presented here suggest that grasshopper ranges should remain stable, if not increase, in the future, grasshoppers might become geographically restricted with the continued loss of grasslands. Some grasses, like *Andropogon gerardii*, are predicted to decline in abundance and extent in the future (Smith et al., 2017), and grasslands are under constant threat of development or agricultural use. Although the abiotic environment might remain favorable to grasshoppers *per se* in the future, there are a number of other factors that will ultimately determine the geographic distribution of North American grasslands in the future.

A second caveat is that ENMs are correlative, and constructed using only two climatic variables. Overwintering temperatures, for example, can set strong constraints on insect distributions (Marshall et al., 2020). However, insect species vary considerably in their dependency on winter temperatures, both among and within life history strategies (i.e., egg vs. nymphal overwintering). For egg wintering species, winter temperature can have a large influence on egg survival and hatching success. The eggs of many species possess low supercooling points, enabling eggs to survive temperatures as low as −25°C (Hao & Kang, 2004), whereas other species can survive cold temperatures only in the presence of an insulating snowpack (Riegart, 1967). Still other species require eggs to be cold stratified for hatching success (Fisher et al., 1996). For nymphal wintering species, the termination of diapause can be trigger by either photoperiod (Ingrisch, 1984) or accumulation of growing degree days (Fisher et al., 1996). Thus, the large variation in how grasshopper species will respond to changes in winter temperature cannot be captured by a general, correlative model. The only means for an ENM to capture such variation is to include winter temperature; however, winter temperatures are highly correlated with spring temperatures (*r* > .90, see Table S2). Thus, winter temperatures are redundant with spring temperatures and add relatively little new information to the model.

Projecting species distributions in future climates remains an important avenue of research. Doing so can inform us of habitat potentially at risk from species invasions (Gong et al., 2020; Kistner‐Thomas, 2019), identify species at risk of collapse (Lemoine, 2015), and pinpoint regions of high priority for conservation (Garzon et al., 2021). In doing so, researchers must carefully account for source of uncertainty. In this study, I accounted for model uncertainty by using nine different ENM estimation techniques, for projection uncertainty by using four separate GCMs, and for scenario uncertainty by using RCP 4.5 and 8.5. My results suggest that phenology might be a good predictor of how insect distributions might change in the future. For North American grasshoppers, early‐season species from cool environments might expand their northern range extent, while late‐season species that are already adapted to hot and dry conditions could experience only modest changes in geographic distribution. My results provide tantalizing evidence that phenology could explain a considerable amount of variation in insect species' ability to respond to climate change.

## CONFLICTS OF INTEREST

The author declares no conflicts of interest.

## AUTHOR CONTRIBUTIONS

**Nathan P. Lemoine:** Conceptualization (equal); data curation (equal); formal analysis (equal); funding acquisition (equal); investigation (equal); methodology (equal); project administration (equal); software (equal); visualization (equal); writing – original draft (equal); writing – review & editing (equal).

## Supporting information

Supplementary Material

## Data Availability

All data, scripts, and figures available on Figshare: 10.6084/m9.figshare.14411048.
