# Supplementary material for: Phenology dictates the impact of climate change on geographic distributions of six co‐occurring North American grasshoppers
Source: Ecol Evol. 2021 Dec 15;11(24):18575–90. doi: 10.1002/ece3.8463 (PMC8717342; doi:10.1002/ece3.8463)

Table S1. Correlation matrix of WorldClim variables. mT_{avg}: Average monthly temperature; mT_{max}: Maximum monthly temperature; mT_{min}: Minimum monthly temperature; mPPT: Average monthly precipitation.

	**mT_{avg}**	**mT_{max}**	**mT_{min}**	**mPPT**
**mT_{avg}**		-	-	-
**mT_{max}**	0.98		-	-
**mT_{min}**	0.98	0.91		-
**mPPT**	0.24	0.20	0.28	

Table S2. Correlation matrix of January T_{min} with T_{min} of all other months. Note the extremely high correlation of January T_{min} with T_{min} of spring months. Bold shows the correlation of winter months to spring months.

	Jan	Feb	Mar	Apr	May	Jun	Jul	Aug	Sep	Oct	Nov	Dec
Jan	1.00	0.99	0.96	0.91	0.86	0.79	0.74	0.80	0.87	0.88	0.94	0.98
Feb	0.99	1.00	0.99	0.95	0.91	0.83	0.78	0.84	0.90	0.91	0.95	0.98
Mar	**0.96**	**0.99**	1.00	0.98	0.95	0.88	0.85	0.90	0.95	0.96	0.97	**0.98**
Apr	**0.91**	**0.95**	0.98	1.00	0.99	0.93	0.91	0.94	0.97	0.97	0.96	**0.94**
May	**0.86**	**0.91**	0.95	0.99	1.00	0.97	0.94	0.96	0.97	0.95	0.92	**0.90**
Jun	0.79	0.83	0.88	0.93	0.97	1.00	0.97	0.96	0.94	0.90	0.85	0.83
Jul	0.74	0.78	0.85	0.91	0.94	0.97	1.00	0.99	0.96	0.92	0.86	0.80
Aug	0.80	0.84	0.90	0.94	0.96	0.96	0.99	1.00	0.99	0.96	0.91	0.87
Sep	0.87	0.90	0.95	0.97	0.97	0.94	0.96	0.99	1.00	0.99	0.96	0.92
Oct	0.88	0.91	0.96	0.97	0.95	0.90	0.92	0.96	0.99	1.00	0.98	0.94
Nov	0.94	0.95	0.97	0.96	0.92	0.85	0.86	0.91	0.96	0.98	1.00	0.98
Dec	0.98	0.98	0.98	0.94	0.90	0.83	0.80	0.87	0.92	0.94	0.98	1.00

Figure S1. Map of raw, unfiltered occurrence records for each of the six species considered here.

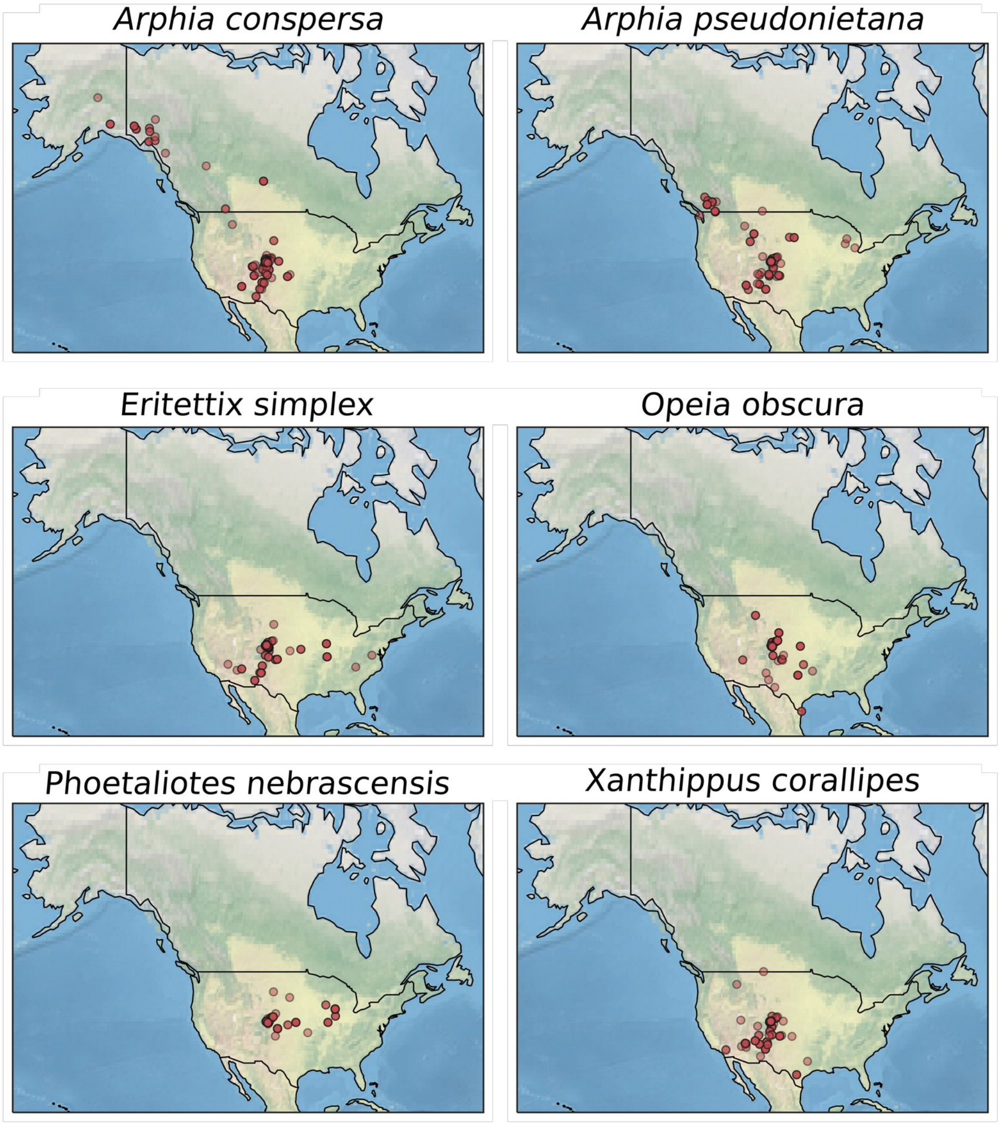

Figure S2. Map of occurrence records in environmental space. Grey points show the unused records, orange points show the environmentally filtered records used in all downstream analyses.

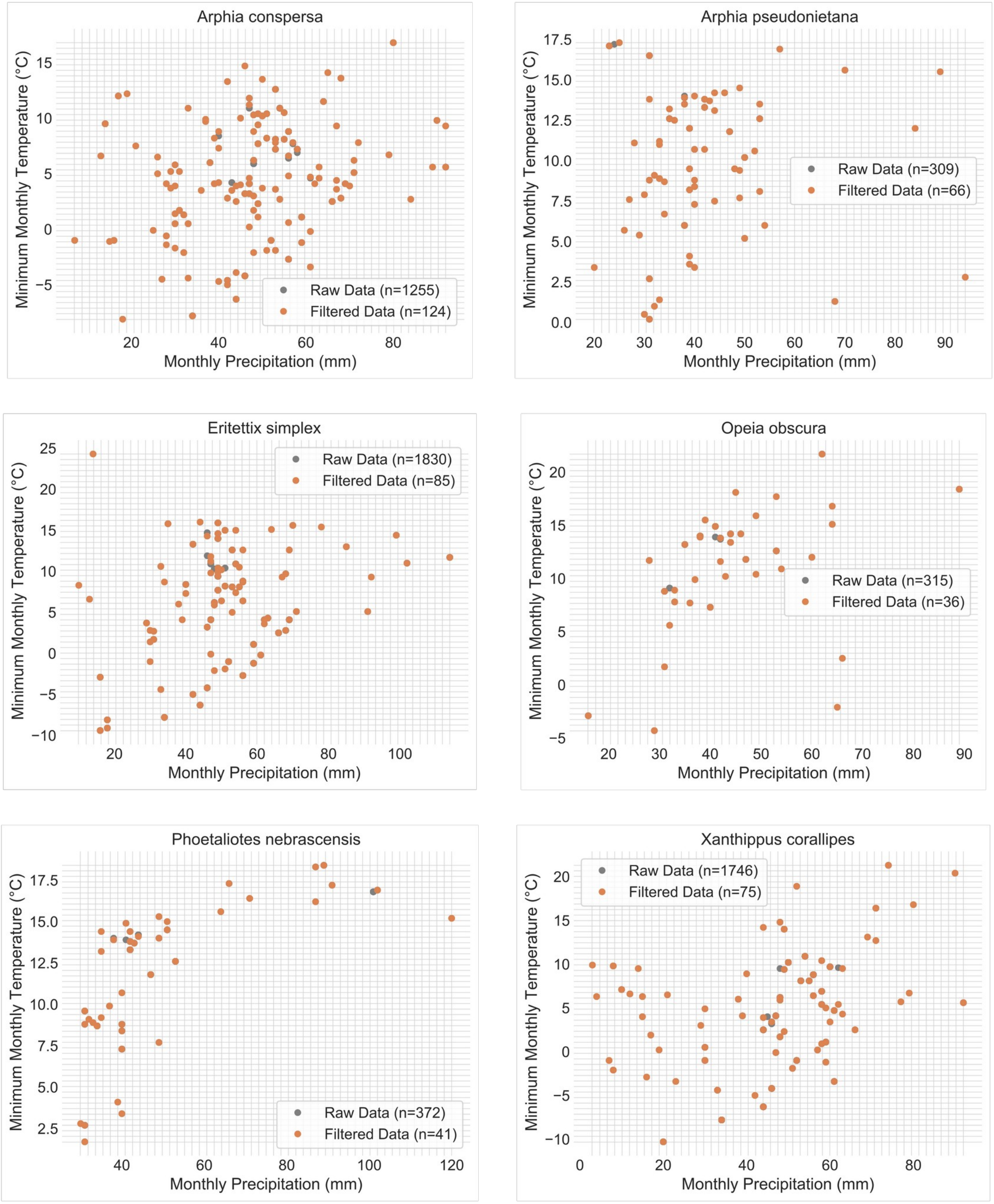

Figure S3. Map of occurrence records after spot-checking and all filtering. These records are the final records used in model building.

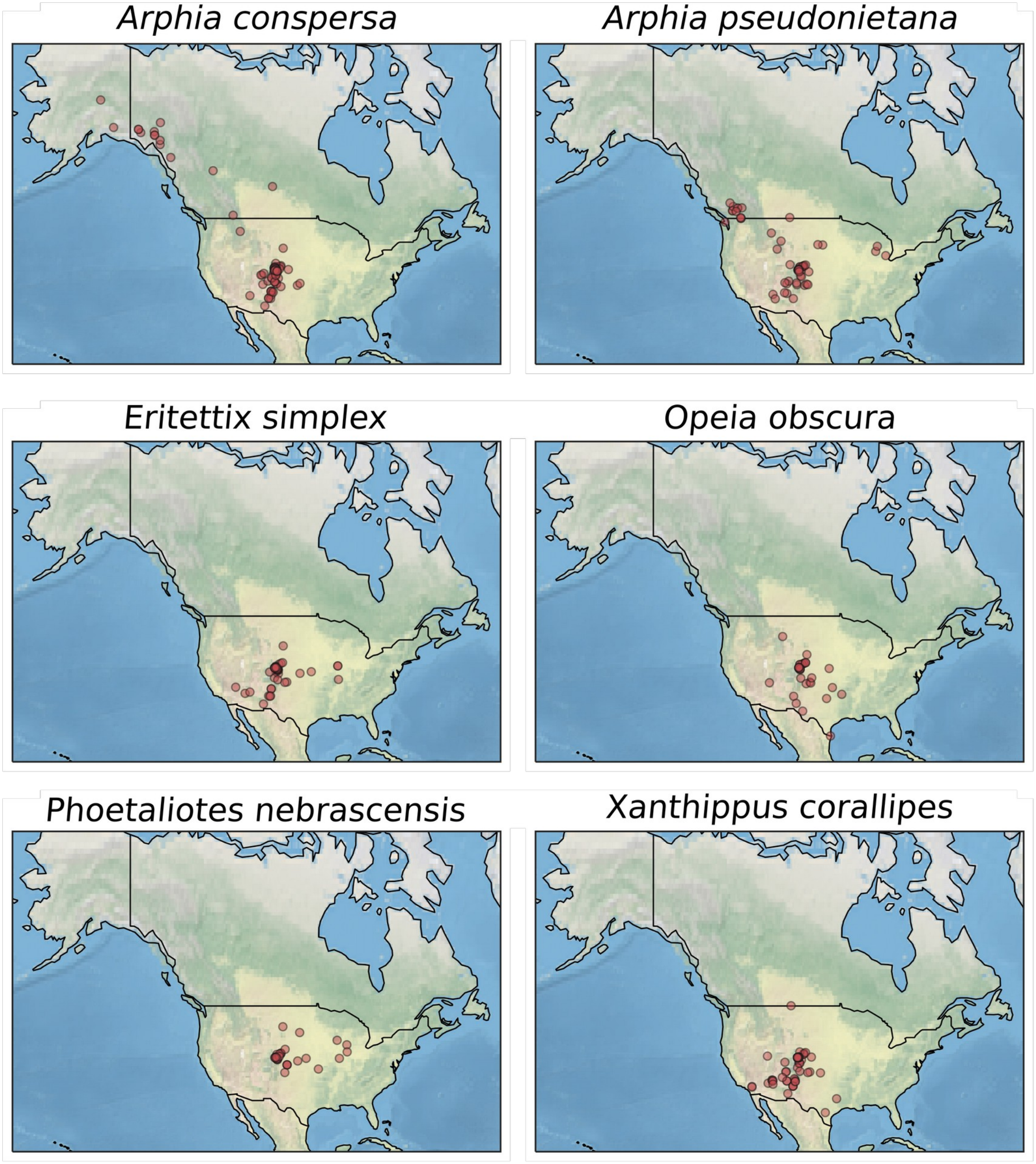

Figure S4. Predicted, current distribution of the early season species *Erittetix simplex* in March, April, and May throughout the Great Plains of North America under current conditions, RCP 4.5, and RCP 8.5. Predictions are the ensemble/stacked averages from the nine different classifiers. The color palette was chosen so that regions where absence is more likely than presence (probability of occurrence < 0.5) are shaded in blue, while regions where presence is more likely than absence (probability of occurrence > 0.5) are shaded in reds. Regions where presence and absence are equiprobable (probability of occurrence ~ 0.5) are shaded in whites/greys.

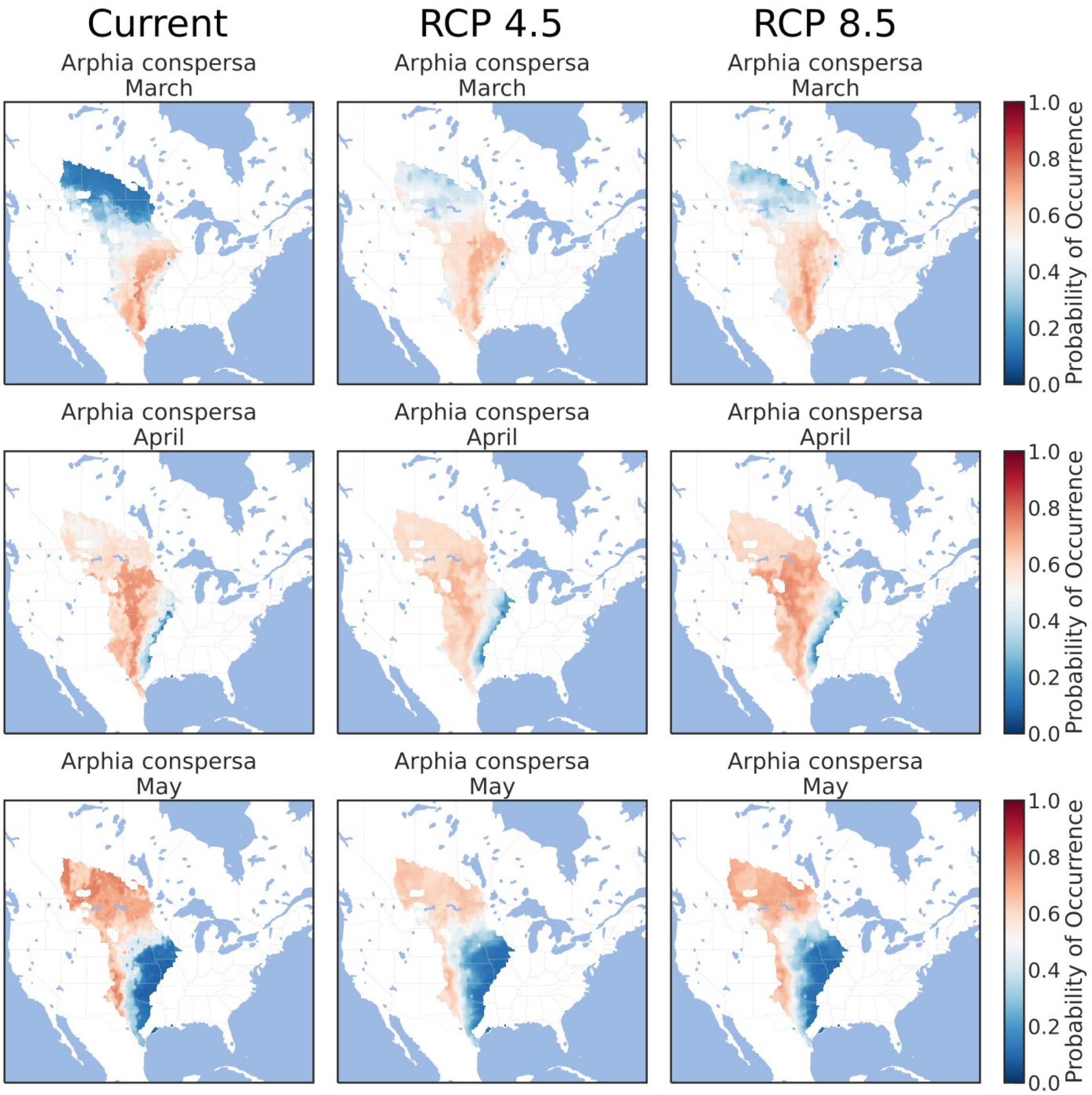

Figure S5. Predicted, current distribution of the early season species *Erittetix simplex* in March, April, and May throughout the Great Plains of North America under current conditions, RCP 4.5, and RCP 8.5. Predictions are the ensemble/stacked averages from the nine different classifiers. The color palette was chosen so that regions where absence is more likely than presence (probability of occurrence < 0.5) are shaded in blue, while regions where presence is more likely than absence (probability of occurrence > 0.5) are shaded in reds. Regions where presence and absence are equiprobable (probability of occurrence ~ 0.5) are shaded in whites/greys.

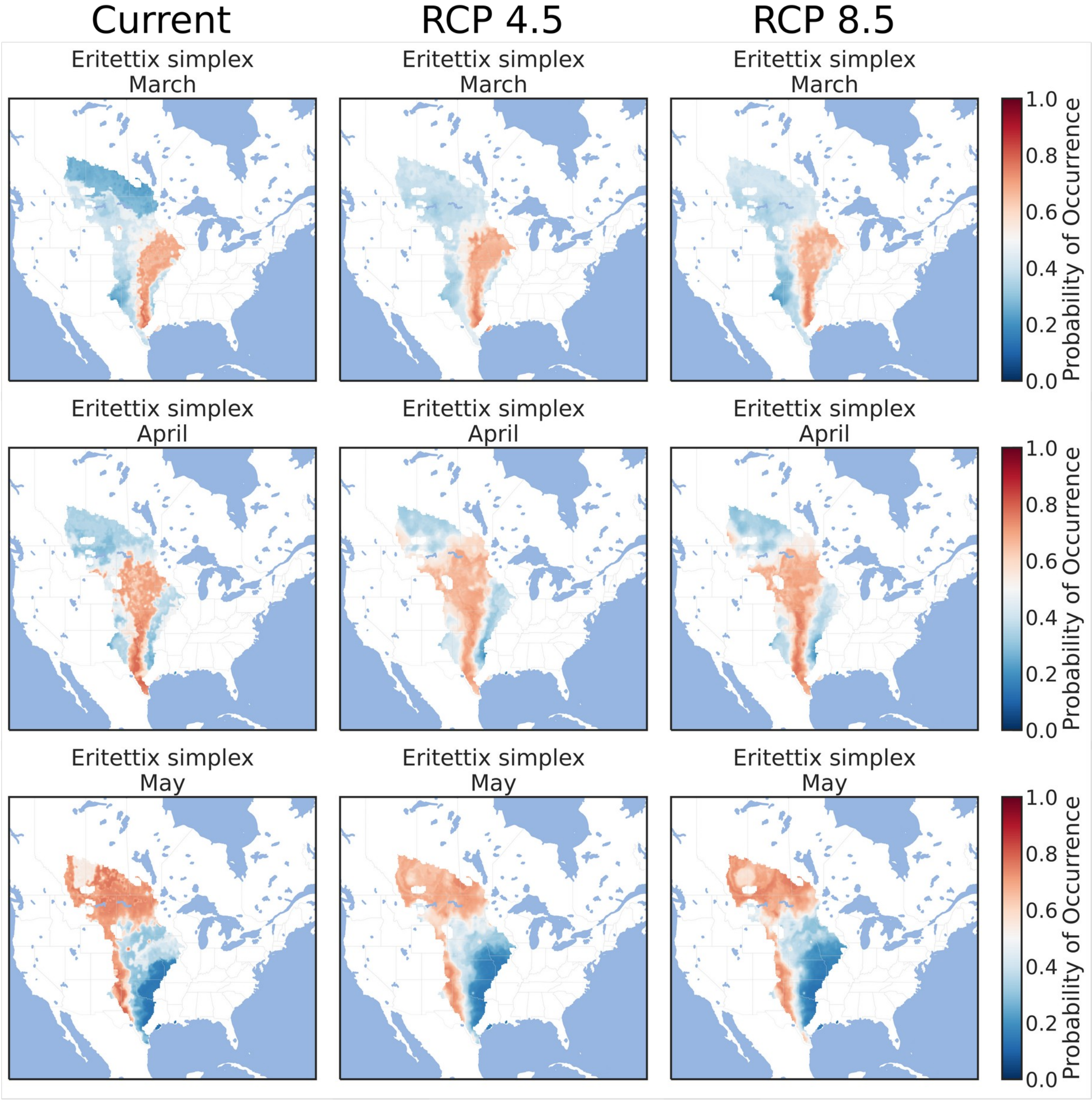

Figure S6. Predicted, current distribution of the early season species *X. corallipes* in March, April, and May throughout the Great Plains of North America under current conditions, RCP 4.5, and RCP 8.5. Predictions are the ensemble/stacked averages from the nine different classifiers. The color palette was chosen so that regions where absence is more likely than presence (probability of occurrence < 0.5) are shaded in blue, while regions where presence is more likely than absence (probability of occurrence > 0.5) are shaded in reds. Regions where presence and absence are equiprobable (probability of occurrence ~ 0.5) are shaded in whites/greys.

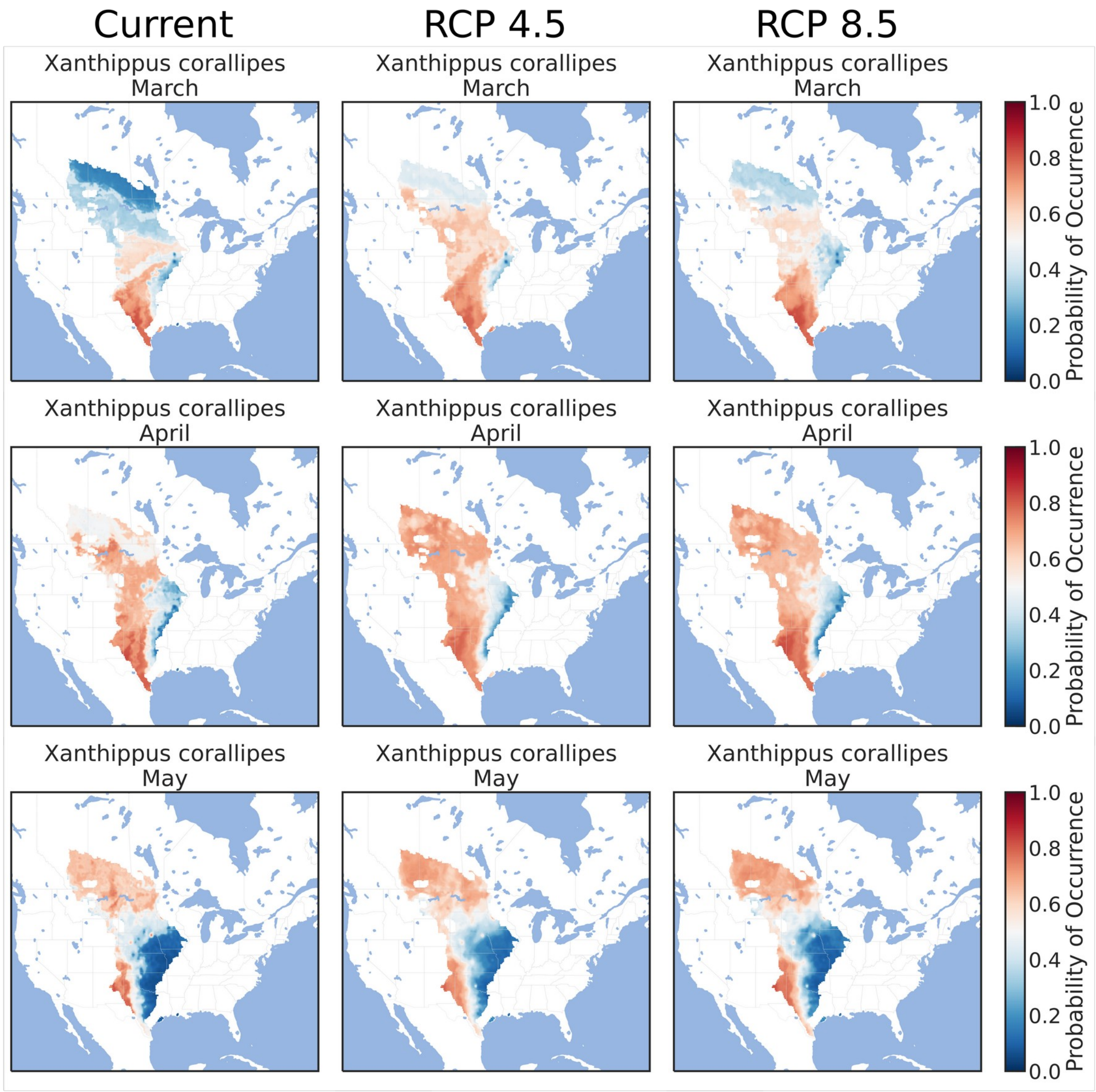

Figure S7. Predicted, current distribution of the early season species *A. pseudonietana* in March, April, and May throughout the Great Plains of North America under current conditions, RCP 4.5, and RCP 8.5. Predictions are the ensemble/stacked averages from the nine different classifiers. The color palette was chosen so that regions where absence is more likely than presence (probability of occurrence < 0.5) are shaded in blue, while regions where presence is more likely than absence (probability of occurrence > 0.5) are shaded in reds. Regions where presence and absence are equiprobable (probability of occurrence ~ 0.5) are shaded in whites/greys.

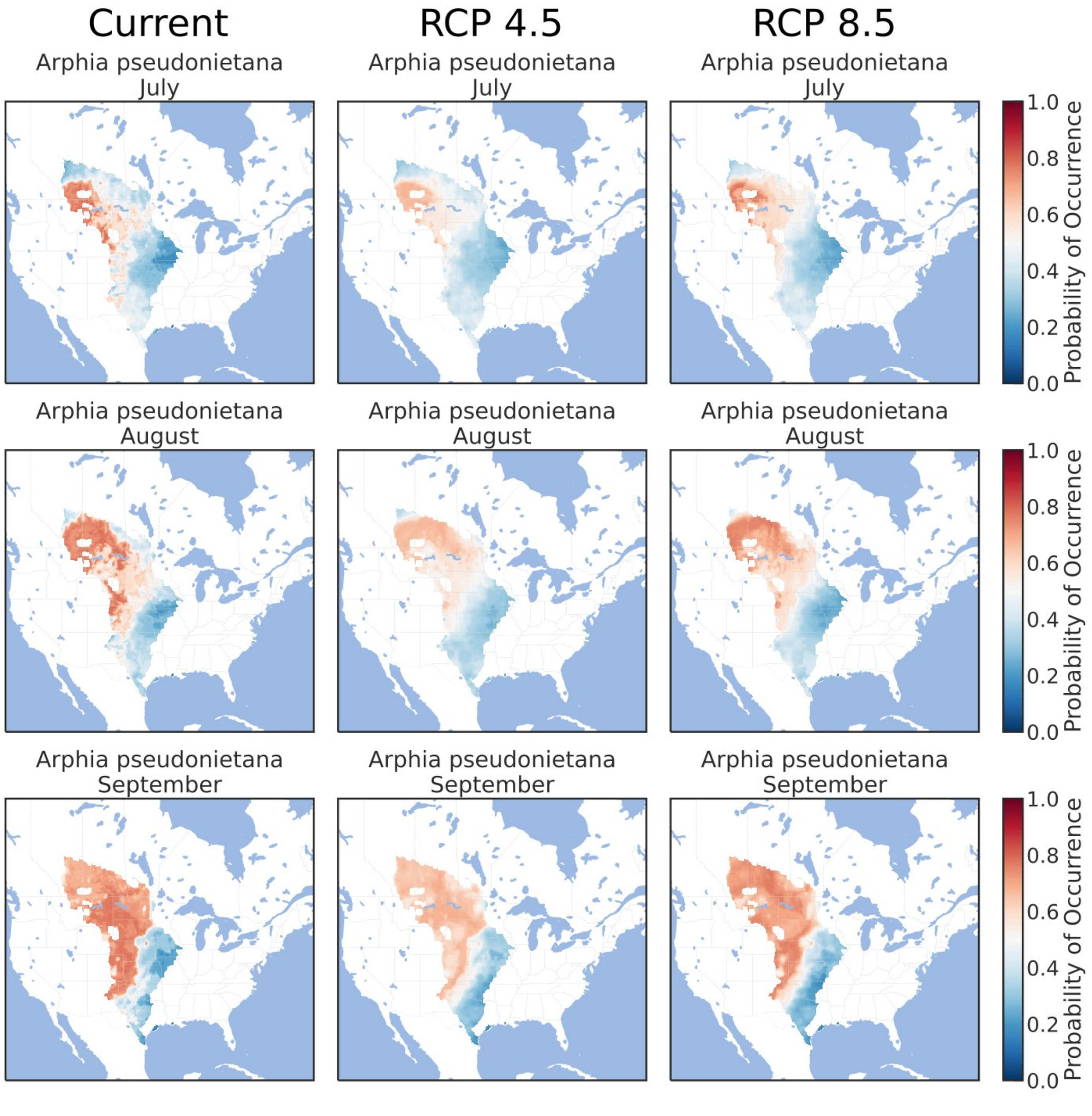

Figure S8. Predicted, current distribution of the late season species *O. obscura* in March, April, and May throughout the Great Plains of North America under current conditions, RCP 4.5, and RCP 8.5. Predictions are the ensemble/stacked averages from the nine different classifiers. The color palette was chosen so that regions where absence is more likely than presence (probability of occurrence < 0.5) are shaded in blue, while regions where presence is more likely than absence (probability of occurrence > 0.5) are shaded in reds. Regions where presence and absence are equiprobable (probability of occurrence ~ 0.5) are shaded in whites/greys.

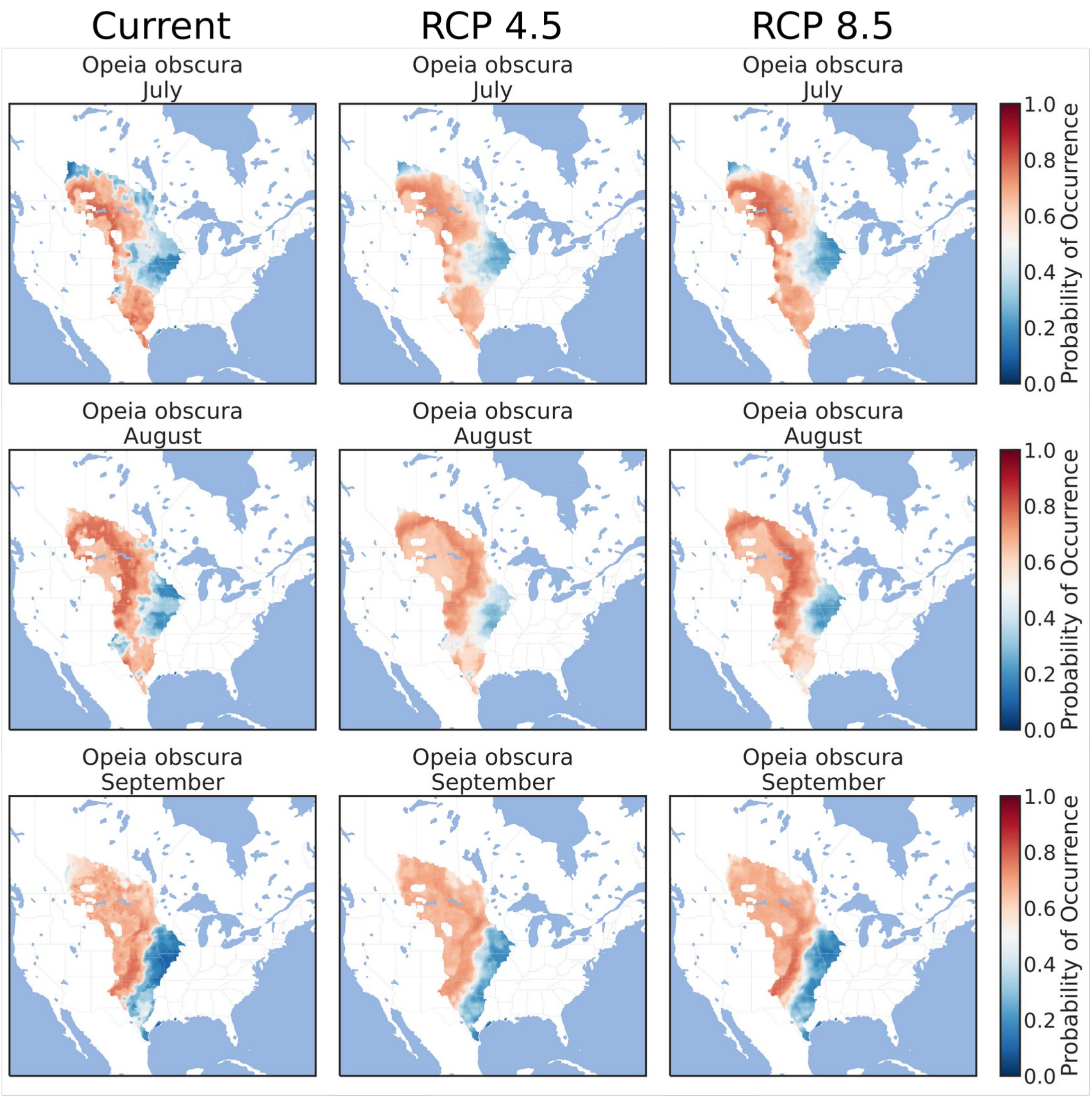

Figure S9. Predicted, current distribution of the late season species *P. nebrascensis* in March, April, and May throughout the Great Plains of North America under current conditions, RCP 4.5, and RCP 8.5. Predictions are the ensemble/stacked averages from the nine different classifiers. The color palette was chosen so that regions where absence is more likely than presence (probability of occurrence < 0.5) are shaded in blue, while regions where presence is more likely than absence (probability of occurrence > 0.5) are shaded in reds. Regions where presence and absence are equiprobable (probability of occurrence ~ 0.5) are shaded in whites/greys.

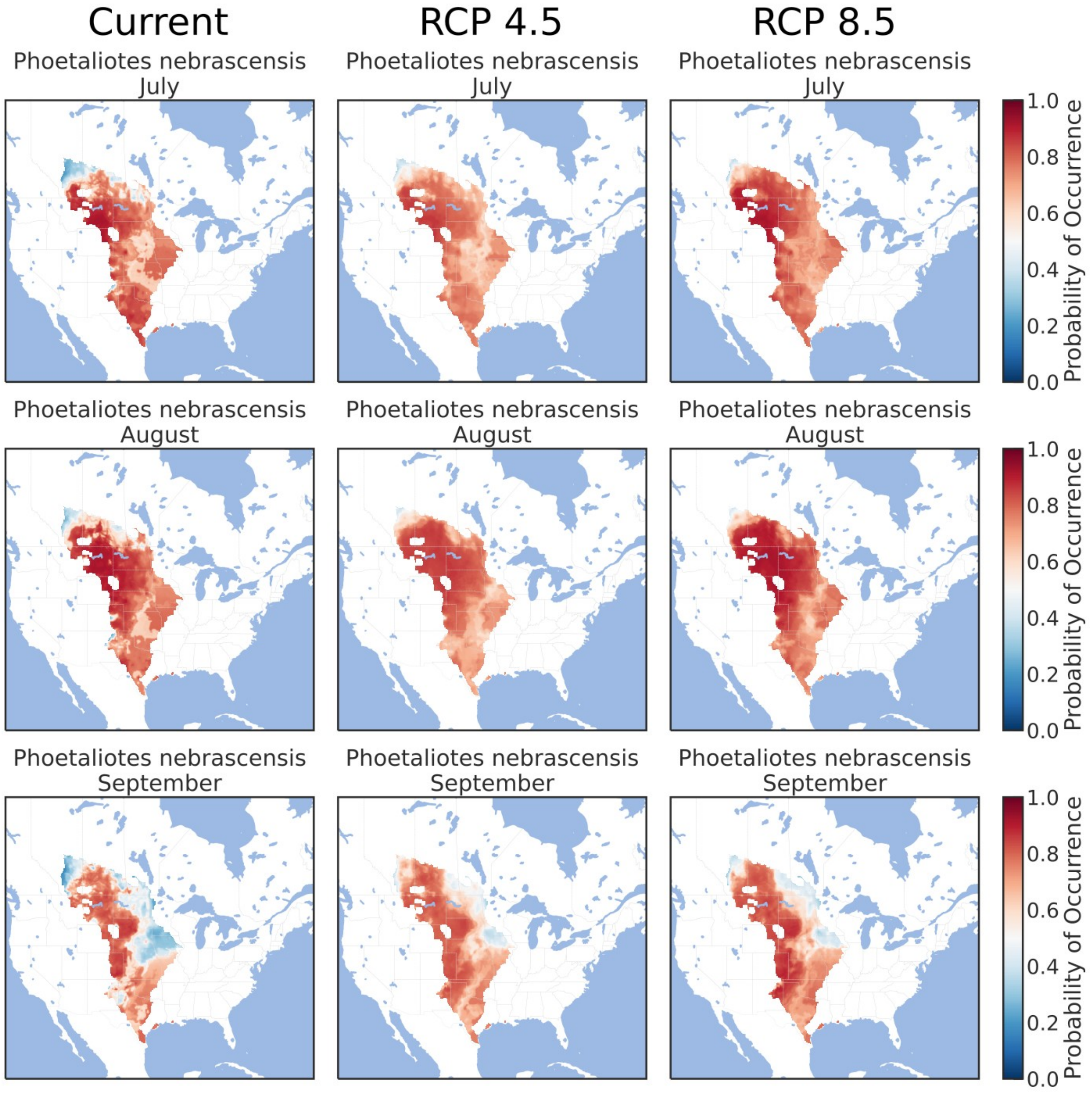

Supplement: Supplementary file 1 — Supplementary Material [file ECE3-11-18575-s001.pdf]
